# Migraine Prevalence, Environmental Risk, and Comorbidities in Men and Women Veterans

**DOI:** 10.1001/jamanetworkopen.2024.2299

**Published:** 2024-03-14

**Authors:** Marianna Gasperi, Nathaniel M. Schuster, Brooke Franklin, Caroline M. Nievergelt, Murray B. Stein, Niloofar Afari

**Affiliations:** 1 Department of Psychiatry and Behavioral Sciences, University of Washington, Seattle; 2 Veteran Affairs (VA) Puget Sound Health Care System, Seattle, Washington; 3 VA Northwest Mental Illness Research, Education and Clinical Center VA Puget Sound Health Care System, Seattle, Washington; 4 Department of Anesthesiology, University of California, San Diego, La Jolla; 5 Department of Psychology, University of Utah, Salt Lake City; 6 Department of Psychiatry, University of California, San Diego, La Jolla; 7 VA San Diego Health Care System, San Diego, California; 8 VA Center of Excellence for Stress and Mental Health, VA San Diego Health Care System, San Diego, California; 9 Herbert Wertheim School of Public Health, University of California, San Diego, La Jolla

## Abstract

**Importance:**

Migraine is a prevalent and debilitating condition that substantially impacts quality of life. Investigating migraine prevalence, associated comorbidities, and potential military service exposures in veterans, focusing on gender differences, is crucial for targeted interventions and management strategies.

**Objective:**

To determine the prevalence of migraine, associated health comorbidities, and potential military service and environmental exposures among men and women US veterans using a large-scale epidemiological sample from the Million Veteran Program (MVP).

**Design, Setting, and Participants:**

This cross-sectional study analyzed self-report survey data from the MVP, a large epidemiological sample of US veterans that was started in 2011 and has ongoing enrollment. Eligible participants were selected from the MVP database in 2023. The study included 491 604 veterans to examine migraine prevalence, health comorbidities, demographic characteristics, military service history, and environmental exposures. Data were analyzed from December 2022 to July 2023.

**Exposures:**

Military service and environmental factors, such as chemical or biological warfare exposure, were considered.

**Main Outcomes and Measures:**

The primary outcome was migraine prevalence among men and women veterans, assessed through self-reported diagnoses. Secondary outcomes included the association between migraine and health comorbidities, demographic characteristics, military service history, and environmental exposures.

**Results:**

Of the 491 604 veterans included in this study, 450 625 (91.8%) were men and 40 979 (8.2%) were women. The lifetime prevalence of migraine was significantly higher in women (12 324 of 40 979 [30.1%]) than in men (36 816 of 450 625 [8.2%]). Migraine prevalence varied by race and ethnicity, with the highest prevalence in Hispanic or Latinx women (1213 of 3495 [34.7%]). Veterans with migraine reported worse general health, higher levels of pain, increased pain interference with work, a higher likelihood of psychiatric and neurological health conditions, and greater lifetime opioid use. Specific aspects of military service, including service post-September 2001 and deployment in Operation Enduring Freedom and Operation Iraqi Freedom, and environmental factors, including Agent Orange, chemical and biological welfare, and antinerve agent pills history, were significantly associated with migraine prevalence.

**Conclusions and Relevance:**

In this cross-sectional study of migraine, the results highlighted gender differences in migraine prevalence and associated health comorbidities among US veterans. The findings emphasized the need for interdisciplinary approaches to migraine management, increased awareness and education efforts, and population-based screening strategies, particularly for women and Hispanic veterans who are at greater risk. Our findings encourage further research into tailored interventions for specific subpopulations and the impact of military service and environmental exposures on migraine and related health conditions.

## Key Points

**Question:**

What is the lifetime prevalence of migraine in US veterans, and how is it associated with comorbidities, environmental risk factors, and service history?

**Findings:**

In this large cross-sectional study of 491 604 US veterans, 8.2% of men and 30.1% of women reported a history of migraine. Veterans with migraine reported significantly worse general health, more pain, and had a higher reported prevalence of neurological and psychiatric conditions, with a noted association to specific environmental exposures during military service.

**Meaning:**

These findings suggest that migraine in veterans is highly prevalent and imposes a significant burden on quality of life; improved screening, tailored interventions, and targeted management strategies are needed to address the unique needs of veterans with migraines.

## Teaser

This cross-sectional study examines the prevalence of migraine, associated health comorbidities, and potential military service exposures among men and women veterans in the US.

## Introduction

Migraine is a common and disabling disorder and a leading cause of neurological primary care visits.^1^ Veterans face a unique challenge, as they have a higher prevalence of migraine and an increased risk for comorbidities, including traumatic brain injury (TBI), posttraumatic stress disorder (PTSD), depression, and anxiety, compared with the general population, which can interact with migraine and worsen symptoms.^2,3^ While the underlying mechanisms of migraine may be similar in veterans and the general population,^4^ the impact of military service and other health comorbidities can result in differences in the prevalence, presentation, and treatment of migraine.^1^ These differences, potentially tied to the physical and psychological stress of military service, are not fully understood. Veterans may also face unique barriers to accessing appropriate treatment for migraine,^5^ including difficulties accessing health care, the stigma associated with seeking care, and limited access to specialized headache clinics. Despite the significant impact of migraine on veterans, there has been limited systematic research^6^ and a lack of understanding of the general epidemiological characterization of migraine in this population across several specific topics.

### Prevalence

Migraine affects up to 12% to 15% of the general population.^7^ However, estimates of migraine prevalence in veterans range from 5% to 10% in general veteran samples^6,8,9^ to 36% in veterans of Operation Enduring Freedom/Operation Iraqi Freedom (OEF/OIF).^10^ Although the causes underlying the high rate of migraine in veterans and varying prevalence rates across service eras^11,12,13^ are not fully understood, various exposures, including TBI, concussion, combat, explosions, and accidents, have been considered possible contributory factors. For example, service during OEF/OIF has been associated with a high risk of TBI^13^ with 485 553 TBIs reported by the US Department of Defense between 2000 and 2023,^14^ which can result in persistent posttraumatic headache, usually presenting with migraine-like phenotype.^15,16^ Similarly, service during the Vietnam and Gulf War eras was associated with various chemical exposures (eg, Agent Orange, anti–nerve agent pills), also considered potential contributors to higher rates of migraine in these veteran cohorts.^17,18^ Evaluating the prevalence of migraine across the veteran population rather than isolated cohorts may elucidate factors affecting prevalence.

### Comorbidities

Migraine, a clinically complex condition, is associated with comorbidities in the general population, including psychiatric disorders and suicide,^19^ sleep disorders,^20^ noncephalic chronic pain conditions (eg, fibromyalgia, temporomandibular disorder, irritable bowel syndrome [IBS], chronic fatigue),^21,22,23,24^ and other domains such as stroke,^25^ epilepsy,^26^ cardiovascular disease,^27^ diabetes and obesity,^28^ and asthma.^29^ Studies have shown that veterans with migraine have more frequent hospitalizations and ED visits than those without migraine.^6^ Migraine poses a considerable burden to the veteran population, manifesting through impairment in daily life, greater health care use, increased comorbidities, and socioeconomic burden.^30,31^ However, the association between migraine and comorbidities is severely understudied and poorly understood in veterans. Research on migraine across a broad range of physical and psychiatric comorbidities in a large, representative sample of veterans from diverse service eras and environmental exposures is limited, and the interplay between migraine and other health conditions in veterans is largely underexplored.

### Gender Differences

Migraine is 2 to 3 times more prevalent and more burdensome^32,33^ in women veterans than men veterans,^32,33,34^ but research on women veteran health, including migraine, continues to lag.^35^ Understanding migraine comorbidities in men and women veterans is critical, as various health comorbidities associated with migraine have been linked with worse migraine phenotypes,^22^ increased migraine severity, increased headache days per month,^23^ and progression to chronic migraine.^36^

### The Present Study

We used a large epidemiological sample of men and women veterans in the Million Veteran Program (MVP) to address these research gaps. We aimed to (1) establish the lifetime prevalence of migraine in veterans of all eras; (2) characterize service history, environmental risk factors, and health comorbidities in those with and without migraine; and (3) examine the role of gender in these associations. By improving our understanding of migraine in this population, our goal is to inform more specialized, effective treatments for migraine and reduce the burden of this debilitating disorder.

## Methods

### Participants and Procedures

Data for this study were from the MVP, a national ongoing research project examining genetic traits, health habits, and environmental factors’ impact on veteran health. The design and recruitment procedures of MVP have been documented previously.^37^ The MVP protocol was approved by the Veterans Affairs (VA) Central Institutional Review Board (cIRB) in 2010, and study enrollment began in 2011. The VA cIRB and the Research and Development Committee at VA San Diego Healthcare System approved the current analyses conducted in 2023. MVP veterans with demographic information and complete data on the baseline survey were included. From 819 417 enrolled veterans, 327 692 were excluded due to missing baseline survey data and 121 due to missing gender, resulting in an analytic sample of 491 604 (59.9%). We followed the Strengthening the Reporting of Observational Studies in Epidemiology (STROBE) reporting guideline.

### Data Sources, Measures, and Analyses

Informed consent was obtained on the day of the MVP study visit, and veterans were asked to complete the self-report baseline survey, as detailed in Gaziano et al.^37^ This survey gathered demographic data, such as race and ethnicity, health, lifestyle, military, medical, and family history. The current study used data on overall health status and pain interference with work throughout the previous 4 weeks, pain level throughout the past week, smoking status, history of diagnosis, military service history, and environmental exposures during military service.

As part of the baseline survey, veterans indicated a history of diagnosis of 75 health conditions from various health domains, including psychiatric, neurologic, hearing and vision problems, infectious disease, respiratory, musculoskeletal, cardiac, and other health conditions. Migraine was assessed as part of self-reported diagnoses. We examined the association with migraine *International Classification of Diseases, Ninth Revision *(*ICD-9*) and *International Statistical Classification of Diseases and Related Health Problems, Tenth Revision *(*ICD-10*) codes (eTable 2 in Supplement 1) in the electronic health record (EHR) to determine the validity of self-reported migraine diagnosis. The tetrachoric correlation between self-reported migraine and lifetime *ICD-9* and *ICD-10* code diagnosis for migraine in the EHR was 0.79 (491 604; 95% CI, 0.79-0.79).

Prescription drug use and alcohol use were obtained from EHR data, part of the VA Corporate Data Warehouse (CDW).^38^ Further information on data types and measures is described in eMethods in Supplement 2).

### Statistical Analysis

All analyses were conducted in R version 4.2.3 (R Project for Statistical Computing).^39^ Demographic data, health conditions, and environmental exposures in veterans with self-reported migraine were reported as mean (SD) or counts with percentages. Men and women were analyzed separately, and differences were assessed using *t* tests for means and 2 sample tests of proportions. To evaluate the presence of migraine across various environmental factors, ORs were adjusted for appropriate covariates, including age, gender, service era, and exposure. For example, odds ratios (ORs) adjusted for Agent Orange included age, gender, and Vietnam War. Because veterans could endorse multiple categories, models were fitted separately for each military branch, service era, and environmental exposure, directly comparing veterans with that experience with veterans without that experience. Logistic regression models with ORs and 95% CIs were used to report the likelihood of association with health conditions for the overall sample (adjusted for age and gender) and across gender (adjusted for age). Further information on data analyses is described in the eMethods in Supplement 2.

The significance level was set at *P* <.001 to account for the large sample size. As described in the eMethods in Supplement 2, to control for type 1 error, false discovery rate (FDR) was used due to the study’s large sample size and multiple tests. FDR significance values (q) are reported as *P* values in the health condition results. Data were analyzed from December 2022 to July 2023.

## Results

### Demographic Characteristics and Health Habits

This study used data from 491 604 veterans (mean [SD] age, 65.1 [12.5] years; 450 625 men [91.8%]; 40 979 women [8.2%]; 63 980 African American veterans [13.3%]; 34 226 Hispanic or Latino/a/x veterans [7.0%]; and 395 265 White veterans [82.3%]). Table 1 displays demographic, health, and lifestyle characteristics for the overall sample and categorized by gender and self-reported migraine diagnosis. Overall, 49 140 veterans (10.0%) reported ever being diagnosed with migraine, with higher lifetime migraine prevalence in women (12 324 of 40 979 [30.1%]) than men (36 816 of 450 625 [8.2%]) (*P* < .001). Veterans reporting migraine were, on average, 8.7 years younger than those without, less likely to be married or cohabiting, had higher education levels, and less likely to report an annual household income less than $60 000.

**Table 1.** Demographic and Health Characteristics of the Total Sample and by Gender and Self-Reported Migraine Diagnosis

Characteristic	Total Sample (N = 491 604)			*P* value^a^	Men (n = 450 625)		*P* value^a^	Women (n = 40 979)		*P* value^a^	*P* value for men vs women^b^
	All^a^	Migraine			Migraine			Migraine			
		Yes	No		Yes	No		Yes	No		
Total participants, No. (%)	491 604 (100)	49 140 (10.0)	442 464 (90.0)	NA	36 816 (8.2)	413 809 (91.8)	NA	12 324 (30.1)	28 655 (69.9)	NA	<.001
Age, mean (SD), y	65.1 (12.5)	57.2 (12.9)	65.9 (12.1)	<.001	59.5 (12.6)	66.6 (11.7)	<.001	50.4 (11.7)	55.4 (13.2)	<.001	<.001
Race and ethnicity^c^											
African American	63 980 (13.3)	7945 (16.6)	56 035 (13.0)	<.001	5440 (15.1)	50 000 (12.4)	<.001	2505 (20.8)	6035 (21.6)	.09	<.001
Hispanic or Latino/a/x	34 226 (7.0)	4543 (9.2)	29 683 (6.7)	<.001	3330 (9.0)	27 401 (6.6)	<.001	1213 (9.8)	2282 (8.0)	<.001	.0100
White	395 265 (82.3)	36 939 (77.0)	358 326 (82.9)	<.001	28 148 (78.3)	337 818 (83.6)	<.001	8791 (72.9)	20 508 (73.2)	.63	<.001
Another race^d^	20 831 (4.3)	3106 (6.5)	17 725 (4.1)	<.001	2340 (6.5)	16 264 (4.0)	<.001	766 (6.4)	1461 (5.2)	<.001	.34
Highest degree completed											
Less than high school	17 374 (3.6)	1061 (2.2)	16 313 (3.7)	<.001	1054 (2.9)	16 272 (4.0)	<.001	7 (0.1)	41 (0.1)	.02	<.001
High school diploma or GED	105 885 (21.9)	7429 (15.3)	98 456 (22.6)	<.001	6743 (18.6)	95 753 (23.5)	<.001	686 (5.6)	2703 (9.6)	<.001	<.001
Some college or bachelor’s	298 383 (61.6)	33 064 (68.3)	265 319 (60.9)	<.001	23 954 (66.1)	245 221 (60.2)	<.001	9110 (74.9)	20 098 (71.2)	<.001	<.001
Master’s or professional, PhD	62 412 (12.9)	6848 (14.1)	55 564 (12.8)	<.001	4491 (12.4)	50 180 (12.3)	.69	2357 (19.4)	5384 (19.1)	.43	<.001
Married, civil union, or cohabitating	294 722 (61.3)	28 258 (58.9)	266 464 (61.6)	<.001	22 931 (63.8)	255 676 (63.2)	.06	5327 (44.2)	10 788 (38.4)	<.001	<.001
Annual household income											
≤$60 000	309 490 (70.7)	29 840 (67.8)	279 650 (71.0)	<.001	22 552 (68.7)	261 864 (71.1)	<.001	7288 (65.5)	17 786 (69.2)	<.001	<.001
$60 000-$99 999	83 140 (19.0)	8975 (20.4)	74 165 (18.8)	<.001	6593 (20.1)	69 366 (18.8)	<.001	2382 (21.4)	4799 (18.7)	<.001	<.001
≥$100 000	45 371 (10.4)	5140 (11.7)	40 231 (10.2)	<.001	3686 (11.2)	37 109 (10.1)	<.001	1454 (13.1)	3122 (12.1)	.008	<.001
BMI	28.7 (5.7)	29.3 (5.9)	28.6 (5.6)	<.001	29.3 (5.7)	28.7 (5.6)	<.001	29.3 (6.5)	28.8 (6.6)	<.001	.400
General health											
Excellent	22 757 (4.7)	1094 (2.2)	21 663 (4.9)	<.001	778 (2.1)	19 896 (4.8)	<.001	316 (2.6)	1767 (6.2)	<.001	<.001
Very good	95 667 (19.6)	6097 (12.5)	89 570 (20.4)	<.001	4241 (11.6)	82 600 (20.1)	<.001	1856 (15.1)	6970 (24.4)	<.001	<.001
Good	177 160 (36.2)	15 784 (32.3)	161 376 (36.7)	<.001	11 278 (30.8)	150 379 (36.5)	<.001	4506 (36.8)	10 997 (38.5)	.001	<.001
Fair	146 213 (29.9)	18 010 (36.8)	128 203 (29.1)	<.001	13 729 (37.5)	121 044 (29.4)	<.001	4281 (34.9)	7159 (25.1)	<.001	<.001
Poor	47 168 (9.6)	7893 (16.1)	39 275 (8.9)	<.001	6600 (18.0)	37 640 (9.1)	<.001	1293 (10.6)	1635 (5.7)	<.001	<.001
Pain interference with work											
Not at all	111 916 (23.0)	4015 (8.2)	107 901 (24.6)	<.001	2983 (8.2)	101 402 (24.8)	<.001	1032 (8.4)	6499 (22.8)	<.001	.25
A little bit	125 252 (25.7)	8671 (17.8)	116 581 (26.6)	<.001	6181 (16.9)	108 561 (26.5)	<.001	2490 (20.3)	8020 (28.2)	<.001	<.001
Moderately	100 918 (20.7)	10 608 (21.8)	90 310 (20.6)	<.001	7787 (21.3)	84 541 (20.6)	.001	2821 (23.0)	5769 (20.3)	<.001	<.001
Quite a bit	105 591 (21.7)	15 683 (32.2)	89 908 (20.5)	<.001	12 033 (33.0)	84 210 (20.6)	<.001	3650 (29.8)	5698 (20.0)	<.001	<.001
Extremely	43 060 (8.8)	9786 (20.1)	33 274 (7.6)	<.001	7518 (20.6)	30 809 (7.5)	<.001	2268 (18.5)	2465 (8.7)	<.001	<.001
10-Point pain scale, past week	4.0 (2.8)	5.6 (2.6)	3.9 (2.8)	<.001	5.6 (2.6)	3.8 (2.7)	<.001	5.6 (2.6)	4.1 (2.8)	<.001	<.001
Smoking status											
Never	139 718 (29.1)	16 921 (35.3)	122 797 (28.4)	<.001	10 979 (30.6)	109 483 (27.1)	<.001	5942 (49.2)	13 314 (47.4)	.001	<.001
Former	251 075 (52.3)	20 480 (42.7)	230 595 (53.4)	<.001	16 748 (46.6)	221 109 (54.7)	<.001	3732 (30.9)	9486 (33.8)	<.001	<.001
Current	89 152 (18.6)	10 596 (22.1)	78 556 (18.2)	<.001	8189 (22.8)	73 280 (18.1)	<.001	2407 (19.9)	5276 (18.8)	.008	<.001
AUDIT-C	1.4 (1.9)	1.2 (1.8)	1.5 (2.0)	<.001	1.3 (1.9)	1.5 (2.0)	<.001	0.9 (1.4)	1.1 (1.6)	<.001	<.001
Antimigraine medication use											
Prevalence	25 883 (5.3)	17 871 (36.3)	8012 (1.8)	<.001	11 089 (30.1)	6193 (1.5)	<.001	6782 (55.0)	1819 (6.3)	<.001	<.001
No. of prescriptions, mean (SD)	18.2 (30.9)	22.9 (34.9)	7.5 (14.6)	<.001	22.9 (35.5)	7.6 (14.6)	<.001	23.1 (33.9)	7.4 (14.9)	<.001	<.001
Opioid medication use											
Prevalence	319 318 (64.9)	37 551 (76.4)	281 767 (63.6)	<.001	27 843 (75.6)	262 074 (63.3)	<.001	9708 (78.8)	19 693 (68.7)	<.001	<.001
No. of prescriptions, mean (SD)	26.2 (48.3)	38.6 (60.0)	24.5 (46.3)	<.001	39.9 (60.9)	24.6 (46.4)	<.001	34.9 (57.3)	23.9 (45.2)	<.001	<.001

Abbreviations: AUDIT-C, Alcohol Use Disorders Identification Test-Concise; BMI, body mass index (calculated as weight in kilograms divided by height in meters squared); GED, general education diploma; NA, not applicable.

^a^ *t* Test for means or 2 sample tests of proportions.

^b^ Comparison between men with migraine and women with migraine.

^c^ Percentage with migraine: African American, 12.4%; Hispanic or Latino/a/x, 13.3%; White, 9.3%, and those reporting another race, 14.9%.

^d^ Includes American Indian or Alaskan Native, Asian, Native Hawaiian or Pacific Islander, and all other races not listed.

Veterans with migraine compared with veterans without migraine had a slightly higher body mass index (BMI, calculated as weight in kilograms divided by height in meters squared; mean [SD], 29.3 [5.9] vs 29.6 [5.6]; *P* < .001) and were more likely to report poor health (16.1% vs 8.9%; *P* < .001) and higher pain levels (mean [SD], 5.6 [2.6] vs mean [SD]. 3.9 [2.8]; *P* < .001). Additionally, 52.3% of veterans with migraine reported quite a bit or extreme pain interference at work compared with 28.1% of veterans without migraine (*P* < .001). Veterans with migraine were more likely to not smoke (35.3% vs 28.4%; *P* = <.001), had lower alcohol use, and had more antimigraine agent (36.3% vs 1.8%; *P* > .001) and lifetime opioid analgesics (76.4% vs 63.6%; *P* > .001) prescriptions.

Across men and women with migraine, the mean (SD) BMI was similar (men, 29.3 [5.7]; women, 28.8 [5.6]; *P* = .40), but smoking status varied with 49.2% of women reporting having never smoked compared with 35.3% of men (*P* < .001). Men had a higher former smoking rate than women (46.6% vs 30.9%; *P* < .001), while current smoking rates were similar. Women reported better health than men (54.5% vs 44.5%), and men reported more pain interference than women (53.6% vs 48.3%) (*P* < .001). Women had more antimigraine medication use than men (55.0% vs 30.1%; *P* < .001) and received slightly more total lifetime antimigraine prescriptions than men (23.1 prescriptions vs 22.9 prescriptions; *P* < .001). Opioid medication use was somewhat higher in women than in men (78.8% vs 75.6%; *P* < .001), but men received more total lifetime prescriptions compared with women (39.9 prescriptions vs 34.9 prescriptions; *P* < .001). Although some differences were small, nearly all variables showed significant differences across gender due to sample size (Table 1).

### Migraine Prevalence

**Figure 1.**
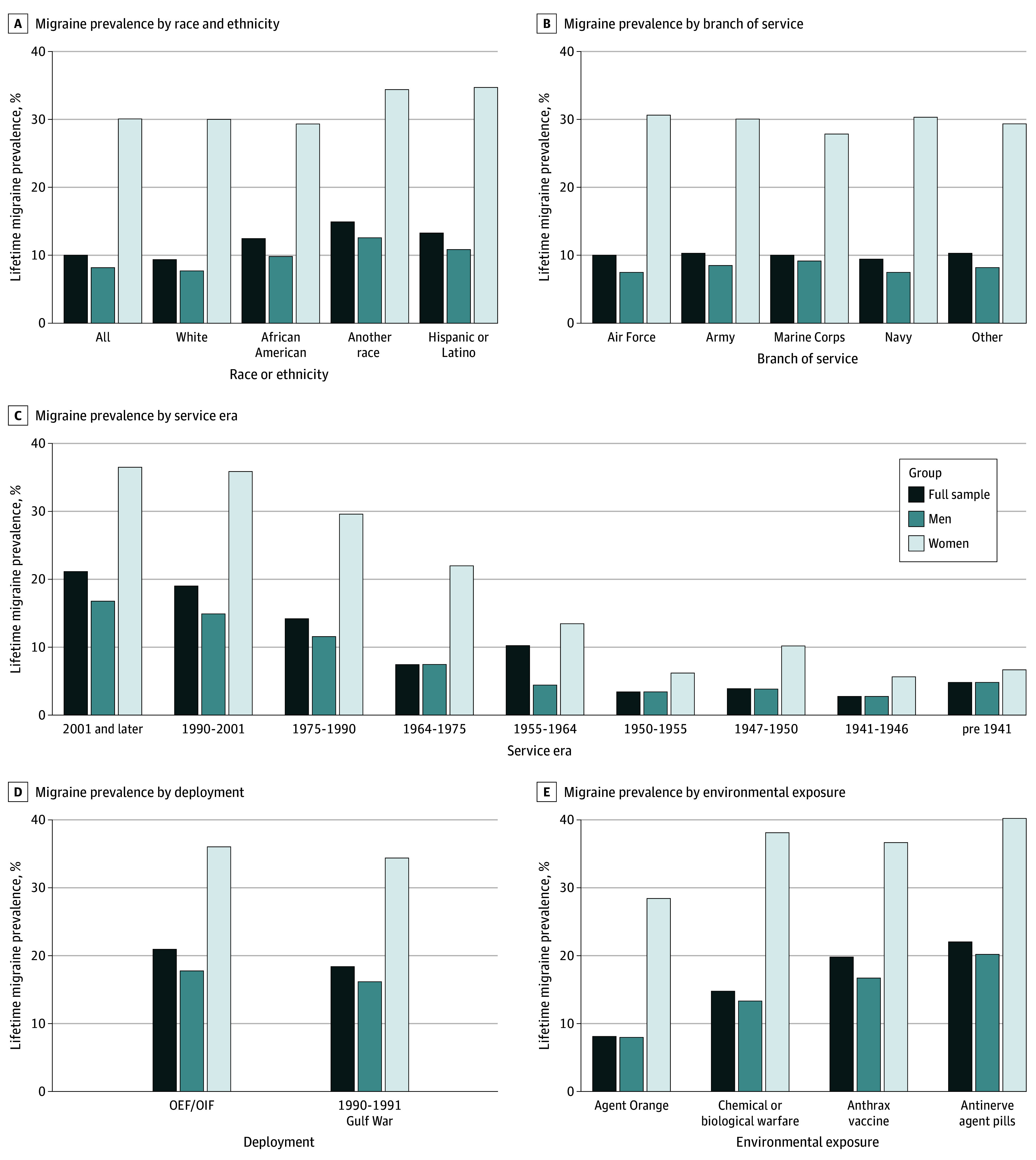
Migraine Prevalence in Men and Women Veterans Across Race or Ethnicity, Branch of Service, Service Era, Deployment, and Environmental Exposure OEF/OIF indicates Operation Enduring Freedom or Operation Iraqi Freedom.

Migraine prevalence (Figure 1) varied by race and ethnicity, service branch, service era (independent of deployment), and deployment. Hispanic or Latinx women had the highest prevalence (1213 of 3495 [34.7%]), and White men had the lowest (28 148 of 365 966 [7.7%]). In the overall sample, higher prevalence was associated with service in other branches (ie, Coast Guard, National Guard, Merchant Marines, National Oceanic and Atmospheric Administration, and Public Health Service) (888 of 8629 [10.3%]), service post-September 2001 (12 502 of 59 144 [21.1%]), and deployment in OEF/OIF (10 154 of 48 529 [20.9%]). For men, higher prevalence was seen in the US Marine Corps (4519 of 49 356 [9.2%]), service post-September 2001 (7716 of 46 030 [16.8%]), and deployment in OEF/OIF (7114 of 40 089 [17.7%]). Higher prevalence in women was seen in the US Navy (2577 of 8498 [30.3%]), service post-September 2001 (4786 of 13 114 [36.6%]), and deployment in OEF/OIF (3040 of 8440 [36.0%]).

Migraine prevalence across environmental exposures (Figure 1) ranged from 4472 of 20 317 (22.0%) for antinerve agent pills to 8445 of 104 435 (8.1%) for Agent Orange. Prevalence was highest for antinerve agent pills at 3728 of 18 466 (20.2%) for men and 744 of 1851 (40.1%) for women, and anthrax vaccine at 9631 of 57 680 (16.7%) for men and 3869 of 10 561 (36.6%) for women.

### Military Service and Environmental Exposure

When controlling for gender and age within the total sample (Table 2), those who served in the US Army (compared with veterans who did not) had slightly increased odds of migraine self-report (adjusted OR [AOR], 1.06; 95% CI, 1.04-1.08). Similarly, those who reported service from August 1990 to August 2001 era, including the Gulf War (AOR, 1.42; 95% CI, 1.39-1.45), and the post-Vietnam era from May 1975 to July 1990 (AOR, 1.31; 95% CI, 1.28-1.33) had higher odds of migraine compared with veterans who did not serve during those eras. Among men (Table 2), service in the US Army had slightly increased odds of migraine self-report (AOR, 1.09; 95% CI, 1.07-1.11), as did service in the August 1990 to August 2001 era, including the Gulf War (AOR, 1.39; 95% CI, 1.35-1.42), and the post-Vietnam era from May 1975 to July 1990 (AOR, 1.30; 95% CI, 1.27-1.33). Among women (Table 2), service in the US Air Force had slightly increased odds of migraine self-report (AOR, 1.10; 95% CI, 1.05-1.16), as did service in the August 1990 to August 2001 era, including the Gulf War (AOR, 1.43; 95% CI, 1.37-1.50), and the post-Vietnam era from May 1975 to July 1990 (AOR, 1.20; 95% CI, 1.14-1.25). Gender interactions (eTable 1 in Supplement 1) with Air Force, Army, Marine Core, Navy, post-2001 service, the Vietnam Era, and deployment in both OEF/OIF and the Gulf War were significant (*P* < .001) in models estimating migraine self-report, with some associations stronger in men and others in women.

**Table 2.** Associations Between Migraine and Military Service and Environmental Exposure for Total Sample and by Gender

Environmental factor	Total sample (N = 491 604)		Men (n = 450 625)		Women (n = 40 979)		*P* value^c^
	AOR (95% CI)	*P* value^a^	AOR (95% CI)	*P* value^b^	AOR (95% CI)	*P* value^b^	
Branch of service							
Air Force	1.01 (0.98-1.04)	.29	0.98 (0.95-1.01)	.30	1.10 (1.05-1.16)	<.001	<.001
Army	1.06 (1.04-1.08)	<.001	1.09 (1.07-1.11)	<.001	0.98 (0.94-1.02)	.40	<.001
Marine Corps	0.97 (0.94-1.01)	.100	0.99 (0.96-1.02)	.600	0.82 (0.75-0.91)	<.001	<.001
Navy	0.91 (0.89-0.94)	<.001	0.90 (0.87-0.92)	<.001	0.99 (0.94-1.05)	.90	<.001
Other^d^	0.85 (0.79-0.91)	<.001	0.85 (0.78-0.92)	<.001	0.85 (0.73-0.99)	.04	.53
Service era							
September 2001 or later (including OEF/OIF)	0.98 (0.95-1.01)	.20	1.00 (0.97-1.04)	.94	0.97 (0.92-1.02)	.25	<.001
August 1990 to August 2001 (including Gulf War)	1.42 (1.39-1.45)	<.001	1.39 (1.35-1.42)	<.001	1.43 (1.37-1.50)	<.001	.254
May 1975 to July 1990	1.31 (1.28-1.33)	<.001	1.30 (1.27-1.33)	<.001	1.20 (1.14-1.25)	<.001	.532
August 1964 to April 1975 (Vietnam era)	1.04 (1.04-1.04)	.49	0.99 (0.97-1.02)	.79	0.90 (0.85-0.96)	.002	<.001
February 1955 to July 1964	0.83 (0.80-0.86)	<.001	0.86 (0.83-0.90)	<.001	0.67 (0.57-0.78)	<.001	.448
July 1950 to January 1955 (Korean War)	0.81 (0.77-0.86)	<.001	0.88 (0.83-0.94)	<.001	0.35 (0.25-0.48)	<.001	.003
January 1947 to June 1950	1.09 (0.95-1.24)	.22	1.17 (1.02-1.33)	.03	0.65 (0.25-1.41)	.32	.693
December 1941 to December 1946 (WWII)	0.86 (0.78-0.94)	<.001	0.94 (0.85-1.04)	.244	0.41 (0.26-0.62)	<.001	.191
November 1941 or earlier	1.35 (0.93-1.89)	.10	1.59 (1.09-2.22)	.01	0.20 (0.01-1.09)	.13	.063
Deployment							
OEF/OIF	1.25 (1.21-1.29)	<.001	1.19 (1.15-1.23)	<.001	0.96 (0.91-1.02)	.20	<.001
1990 to 1991 Gulf War	1.55 (1.50-1.59)	<.001	1.58 (1.53-1.63)	<.001	1.31 (1.22-1.39)	<.001	<.001
Environmental exposure							
Agent Orange^e^	1.60 (1.55-1.65)	<.001	1.68 (1.62-1.73)	<.001	1.39 (1.16-1.66)	<.001	.88
Chemical or biological warfare^f^	2.19 (2.12-2.26)	<.001	2.31 (2.24-2.39)	<.001	1.64 (1.51-1.78)	<.001	<.001
Anthrax vaccine^f^	1.50 (1.46-1.55)	<.001	1.65 (1.59-1.71)	<.001	1.15 (1.08-1.22)	<.001	<.001
Antinerve agent pills^f^	2.14 (2.05-2.22)	<.001	2.25 (2.16-2.35)	<.001	1.50 (1.36-1.66)	<.001	<.001

Abbreviations: AOR, adjusted odds ratio; OEF/OIF, Operation Enduring Freedom or Operation Iraqi Freedom.

^a^ Adjusted for age and gender.

^b^ Adjusted for age.

^c^ *P *value for gender by environmental factor interaction. Significance value for the interaction between gender and environmental exposure factor, adjusted for age.

^d^ Other branch of service includes Coast Guard, National Guard, Merchant Marines, National Oceanic and Atmospheric Administration, Public Health Service.

^e^ Adjusted for age and Vietnam War.

^f^ Adjusted for age, service during the Gulf War, and service after September 2001.

All environmental exposures assessed were associated with increased odds of migraine self-report, even when adjusting for age, gender, and relevant service history (Table 2). The highest odds were seen for chemical and biological warfare exposure (AOR, 2.19; 95% CI 2.12- 2.26) and lowest for Anthrax vaccine (AOR, 1.50; 95% CI, 1.46-1.55). Significant associations between all environmental exposures and migraine were found in men (Table 2), with the strongest for chemical or biological warfare (AOR, 2.31; 95% CI, 2.24-2.39). Similarly, all environmental exposures were also associated with migraine in women, with the strongest for chemical or biological warfare (AOR, 1.64; 95% CI, 1.51-1.78). Gender interactions were significant (stronger in men) for all exposures except Agent Orange.

### Self-Reported Health Conditions

**Figure 2.**
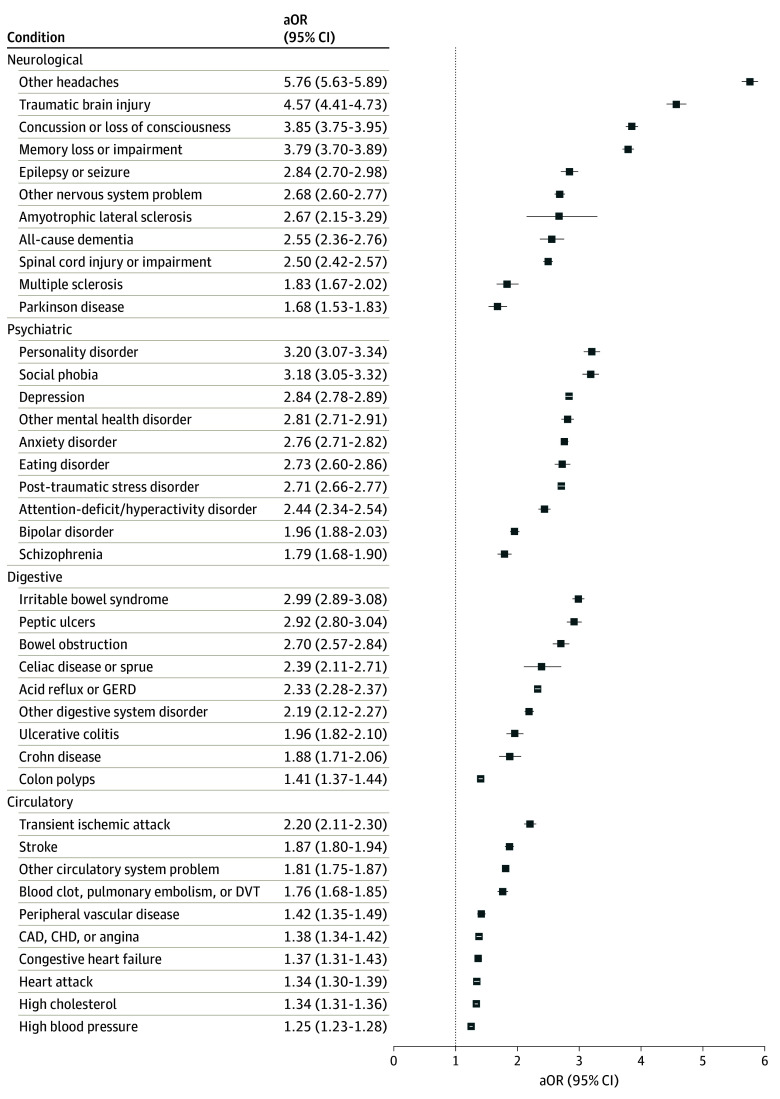
Self-Reported Neurological, Psychiatric, Digestive, and Circulatory Conditions Across Migraine Status for the Overall Sample AOR indicates adjusted odds ratio; CAD, coronary artery disease; CHD, congenital heart defects; DVT, deep vein thrombosis; GERD, gastroesophageal reflux disease.

**Figure 3.**
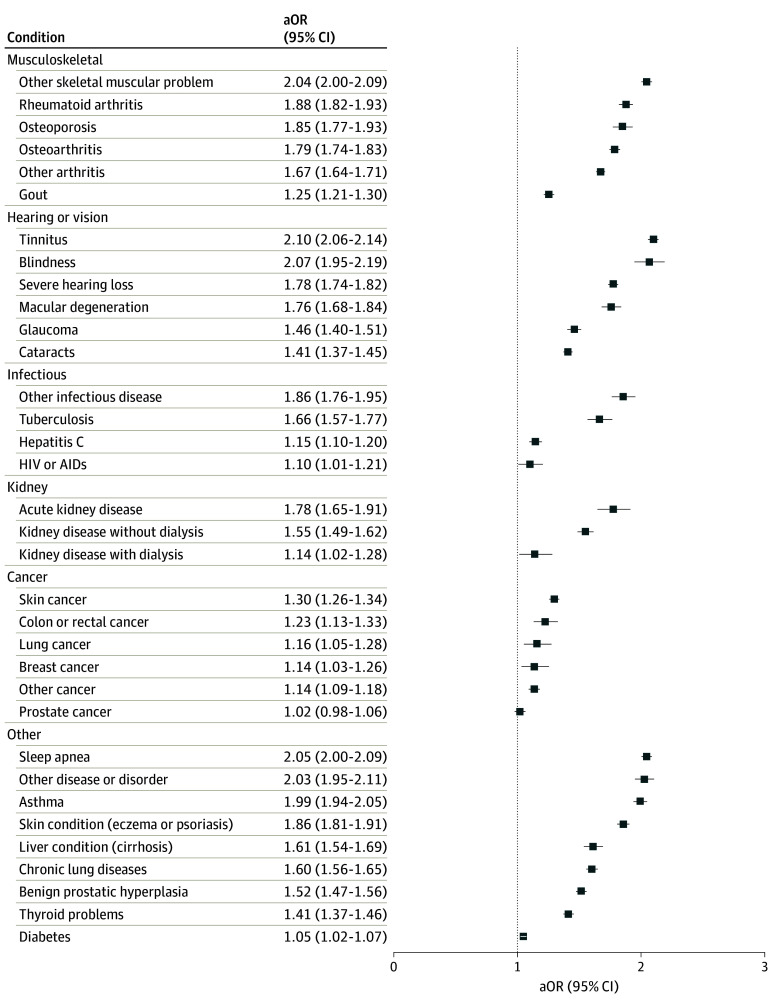
Self-Reported Musculoskeletal, Hearing and Vision, Infectious, Kidney, Cancer, and Other Conditions Across Migraine Status for the Overall Sample AOR indicates adjusted odds ratio.

eTable 1 in Supplement 1 provides the prevalence of health conditions in the overall sample and by migraine status and by migraine status and gender. Within the total sample, migraine was associated with most health conditions examined when adjusted for age and gender (Figure 2 and Figure 3). Among men (eFigure 1 and eFigure 2 in Supplement 2), the strongest associations were with other headaches (AOR, 6.44; 95% CI, 6.28-6.60), TBI (AOR, 4.82; 95% CI, 4.65-5.00), concussion or loss of consciousness (AOR, 4.05; 95% CI, 3.94-4.16), and memory loss or impairment (AOR, 3.95; 95% CI, 3.84-4.05). Personality disorder, social phobia, other mental health disorder, eating disorder, depression, and IBS all had odds over 3.00 (*P* < .001). Among women, the strongest associations were with other headaches (AOR, 3.36; 95% CI, 3.19-3.55), TBI (AOR, 2.92; 95% CI, 2.80-3.05), concussion or loss of consciousness (AOR, 2.89; 95% CI, 2.70-3.09), and memory loss or impairment (AOR, 2.88; 95% CI, 2.69-3.09) (eFigure 1 and eFigure 2 in Supplement 2). Notably, while the associations were slightly weaker in women than men, the overall pattern remained consistent. Significant interactions between migraine and gender were present (with most associations stronger in men) for over half of the evaluated conditions (indicated by asterisks in eFigure 1 and eFigure 2 in Supplement 2), including all psychiatric disorders.

## Discussion

We used data from the MVP to assess the lifetime prevalence of migraine among US veterans, considering gender, service eras, environmental risk factors, and health comorbidities. Lifetime migraine prevalence was 8.2% in men and 30.1% in women, with the highest rates among Hispanic or Latinx women and in more recent service eras. Veterans with migraine were more likely to report additional psychiatric, neurological, and digestive conditions. Importantly, our results showed higher migraine prevalence in the context of specific aspects of military service and environmental exposures. These findings shed light on the complex association between migraine, gender, and military service–related environmental exposures, particularly among the often-understudied demographic of women veterans. In deepening the insights of prior work,^6^ our research further elucidates the interplay between migraine, gender, diverse lifetime comorbidities, and various environmental exposures.

Our results aligned with previous research.^6^ The higher prevalence of migraine among women, especially Hispanic or Latinx women, supported earlier findings of increased burden of migraine in these populations.^40^ The higher prevalence of migraine in women than men echoes previous research and may be attributable to endocrine, genetic, and environmental influences.^41^ The increased prevalence of migraine in veterans, especially in more recent military service eras, supports the evolving interplay between contemporary service conditions and migraine incidence. Additionally, our results support the significant comorbidity of migraine with psychiatric, neurological, and digestive health issues, reinforcing the importance of integrated health care approaches in managing these comorbidities.

We provide novel insights into the association between migraine and specific environmental exposures associated with military service. Our results suggest that certain occupational or environmental factors, such as Agent Orange and chemical and biological welfare, are associated with migraine in veterans. The increased prevalence of migraine suggests that such exposures may elevate the risk of associated conditions with potential gender-specific impacts. Men may exhibit greater vulnerability to certain service-related environmental triggers, either through genetic predisposition or environmental exposure. Our results underscore the potential differential impact of these exposures on men and women, emphasizing the need for more gender-focused research and health care strategies to address these disparities. Recognizing the impact of environmental exposures, the VA’s implementation of the Honoring Our Promise to Address Comprehensive Toxics (PACT) Act of 2022 environmental exposure screening law is timely and commendable. PACT mandates comprehensive health evaluations for all veterans to identify and address potential environmental exposures and expands VA health care and benefits for veterans exposed to burn pits, Agent Orange, and other toxins. Such screening may help identify the factors contributing to migraine and other health conditions, ultimately enabling appropriate treatment.

The association between migraine and quality of life was evident, as veterans with migraine reported diminished general health, increased pain levels, and interference with work. The increased use of opioid medications among veterans who reported migraine compared with those who did not highlights the importance of guidelines-based migraine care for veterans.^42,43,44^ Interdisciplinary approaches to migraine management, encompassing pharmacological and nonpharmacological treatments, are crucial for addressing the diverse needs of impacted individuals.^45,46^ Because comorbid pain, neurological, psychiatric, and digestive disorders can affect the association between migraine, health characteristics including pain and opioid use, a multifaceted approach to migraine research to understand these complicated associations and to clinical management is essential. Enhanced awareness and education for health care clinicians and the public can promote earlier detection of migraine symptoms, leading to better diagnoses and patient outcomes.^47^ These efforts can also mitigate the impact of migraine and associated comorbidities, contributing to more efficient health care use. To ensure effective treatment and prevention, specialized screening and interventions should be considered for specific groups, such as women and Hispanic or Latinx veterans.

Finally, the comorbidity of migraine with other health conditions, particularly psychiatric and neurological disorders, was consistent with previous work^22,24,48,49,50,51^ and highlights the importance of screening and addressing cognitive and mental health concerns in veterans with migraine rather than regarding migraine as an adverse effect of other conditions. In line with this, comprehensive neurological evaluations for veterans with migraine are essential, given the strong association with conditions like TBI. The higher prevalence of digestive system problems, such as IBS and others, in veterans with migraine warrants further investigation.

### Limitations

This study has limitations. Using data from a single source (the MVP) may limit generalizability due to potential biases of the sample, including demographic and clinical characteristics. Diagnoses were based on self-report rather than the more rigorous *International Classification of Headache Disorders 3rd edition* criteria or a validated screener, which might lead to underdiagnosis. This self-report approach can introduce recall or social desirability bias, and recall can be influenced by an individual’s health status, even though a strong correspondence with medical record data was observed. The cross-sectional design restricts establishing causality or directionality, necessitating longitudinal studies to elucidate temporal associations and potential causal pathways. Detailed clinical or diagnostic information on migraine was not available; therefore, future studies can replicate these findings with clinical samples using clinical and diagnostic information.

## Conclusions

In this cross-sectional study of the association between migraine, environmental exposures, and various health conditions in US veterans, findings underscored the need for more research on migraine etiology, diagnosis, and management. Our results suggested that comprehensive and targeted migraine management strategies should be prioritized to address the unique needs of different veteran populations and at-risk groups. This work emphasized that collaborative efforts among health care clinicians, researchers, and policy makers within the VA health care system are essential to improve the diagnosis and treatment of migraine.

## Notes

Supplement 1. **eTable 1.** Self-Reported Prevalence of Health Conditions for Full Sample and Across Migraine **eTable 2.** *International Classification of Diseases, Ninth Revision and Tenth Revision* Migraine Disorder Codes and Crosswalk Used in Self-Report Validation

Supplement 2.
eMethods.

eAcknowledgments.
**eFigure 1.** Self-Reported Neurological, Psychiatric, Digestive, and Circulatory Conditions Across Migraine Status for Men and Women **eFigure 2.** Self-Reported Musculoskeletal, Hearing and Vision, Infectious, Kidney, Cancer, and Other Conditions Across Migraine Status for Men and Women
eReference.

Supplement 3.
Data Sharing Statement
