# Supplementary material for: Migraine Prevalence, Environmental Risk, and Comorbidities in Men and Women Veterans
Source: JAMA Netw Open. 2024 Mar 14;7(3):e242299. doi: 10.1001/jamanetworkopen.2024.2299 (PMC10940970; doi:10.1001/jamanetworkopen.2024.2299)
Supplement: Supplement 2. — eMethods. eAcknowledgments. eFigure 1. Self-Reported Neurological, Psychiatric, Digestive, and Circulatory Conditions Across Migraine Status for Men and Women eFigure 2. Self-Reported Musculoskeletal, Hearing and Vision, Infectious, Kidney, Cancer, and Other Conditions Across Migraine Status for Men and Women eReference. [file jamanetwopen-e242299-s002.pdf]

Supplemental Online Content

Gasperi M, Schuster NM, Franklin B, Nievergelt CM, Stein MB, Afari N. Migraine prevalence, environmental risk, and comorbidities in men and women veterans. *JAMA Netw Open*. 2024;7(3):e242299. doi:10.1001/jamanetworkopen.2024.2299

eMethods.
eAppendix.
eTable 1. Self-reported Prevalence of Health Conditions for Full Sample and Across Migraine

eTable 2. *International Classification of Diseases, Ninth Revision and Tenth Revision* Migraine Disorder Codes and Crosswalk Used in Self-report Validation

eFigure 1. Self-Reported Neurological, Psychiatric, Digestive, and Circulatory Conditions Across Migraine Status for Men and Women

eFigure 2. Self-Reported Musculoskeletal, Hearing and Vision, Infectious, Kidney, Cancer, and Other Conditions Across Migraine Status for Men and Women
eReferences

This supplemental material has been provided by the authors to give readers additional information about their work.

© 2024 Gasperi M et al. *JAMA Network Open.*

eMethods: Supplemental Methods

Data Sources and Measures

The current study utilized the MVP questionnaire and electronic health record (EHR) data, including VA's Corporate Data Warehouse (CDW) pharmacy records. [1] All survey data, except alcohol and prescription drug use, were obtained from the MVP Baseline Survey for analysis.

Baseline Survey

Veterans were asked to complete the MVP Baseline Survey at enrollment. The Baseline Survey has been described previously and can be found in the supplementary materials within Gaziano et al. [2] Briefly, the self-report survey was developed to collect demographic, family pedigree, health status, lifestyle habits, military experience, medical history, family history of specific illnesses, and physical features. Specific data types are described in more detail below.

Demographic variables. Age at assessment, self-reported race, ethnicity, marital status, highest level of education completed, and household income were reported at time of survey completion. Race and ethnicity were assessed to characterize the sample demographics.

Health characteristics. Overall health status (rated on a five-point Likert scale from “excellent” to “poor”) and pain interference with work (ranging from “not at all” to “extremely”) were derived from The Veterans RAND 12-Item Health Survey (VR-12) [3] (question 1 and question 5, respectively) which assesses health function over the past four weeks. Pain level during the past week was assessed using a 10-point pain scale, with 0 indicating no pain and 10 indicating "pain as bad as you can imagine." [4] Ratings of 0 correspond to no pain, 1 to 3 to mild pain, 4 to 6 to moderate pain, and 7 to 10 to severe pain.

Body mass index (BMI) was calculated from height and weight reported on the Baseline survey. Smoking status (never/former/current) was obtained at the time of survey completion based on answers to the following two questions: "In your lifetime, have you smoked a total of at least 100 cigarettes?" and “Do you now smoke cigarettes?" (Yes/No as response alternatives).

Self-Reported Lifetime Diagnosis of Psychiatric, Neurological, and Health Conditions. As part of the Baseline Survey, participants indicated a history of diagnosis of 75 health conditions from various health domains, including psychiatric, neurological (including migraine headache), hearing and vision problems, infectious disease, respiratory, musculoskeletal, cardiac, and other health conditions. Participants were instructed, "Please tell us if you have been diagnosed with the following conditions," followed by a list of conditions, thus, the self-reported health issues should be regarded as lifetime ("ever") conditions. The tetrachoric correlation between self-reported migraine and ICD9/10 codes for migraine in the EHR was 0.79 (n=491,604; 95% CI, 0.79-0.79).

Military Service History and Environmental Exposure.

Branch of Service. Uniformed services exposure assessed on the Baseline Survey included non-mutually exclusive categories of Air Force, Army, Marine Corps, and Navy

© 2024 Gasperi M et al. *JAMA Network Open.*

service. Additional categories included Coast Guard, National Guard, Merchant Marines, National Oceanic and Atmospheric Administration (NOAA), and Public Health Service and were combined into "other service." Veterans could endorse multiple options that were relevant to them.

Era of Service. The era of service was assessed across the following non-mutually exclusive categories: September 2001 or later, August 1990 to August 2001 (including Gulf War), May 1975 to July 1990, August 1964 to April 1975 (Vietnam era), February 1955 to July 1964, July 1950 to January 1955 (Korean War), January 1947 to June 1950, December 1941 to December 1946 (WWII), November 1941 or earlier. Veterans could endorse multiple options that were relevant to them.

Deployment. The survey assessed the 1990-91 Gulf War and OEF/OIF deployment.

Environmental exposure. Environmental exposures during military service were assessed using the Structured Neurotoxicant Assessment Checklist (SNAC) 5, which included Agent Orange exposure, chemical or biological warfare exposure, anthrax vaccine, and anti-nerve agent pills.

VA Electronic Health Record

Prescription drug and alcohol use were derived from the VA EHR data part of the CDW. VA drug classification system classes (VA grouping of drugs to help identify products with similar mechanisms and effects) CN101 and CN105 were used to identify the lifetime use of opioid analgesics and antimigraine agent medications, respectively, from VA EHR outpatient medication data.

Alcohol risk was ascertained using the Alcohol Use Disorders Identification Test-Concise (AUDIT-C) [6] alcohol screen within one year of the Baseline Survey data collected as part of routine clinical care. Because the AUDIT-C is completed intermittently, the score closest to the Baseline Survey administration was used for each participant, with a mean of 3.1 months (SD = 2.5) between the two.

Statistical Analyses

Prevalence of health conditions for the full sample and across migraine comparison was conducted using logistic regression models with adjusted ORs and 95% CIs and were adjusted for age and gender.

Migraine prevalence across different types of military service branches, service era, deployment, and environmental exposure was evaluated using separate logistic regression models with adjusted ORs and 95% CIs adjusted for relevant covariates, age, and gender. Specifically, the deployment history in OEF/OIF and the 1990-91 Gulf War analyses was adjusted for age and gender. Exposure to Agent Orange analysis was adjusted for age, gender, and Vietnam War service era. History of chemical or biological warfare exposure, Anthrax vaccine, and anti-nerve agent pills analyses were adjusted for age, gender, Gulf War, and September 2001 or later service eras. Similarly, logistic regression models with adjusted ORs and 95% CIs were used to report the likelihood of migraine headache in men and women. P-

© 2024 Gasperi M et al. *JAMA Network Open.*

values for the interaction between gender and environmental exposure in the overall logistic regression model, with significance set at <.001 to account for the large sample size, were used to assess differences in migraine headache by sex and various environmental exposures.

Due to the large sample size of the current study and multiple tests conducted, the false discovery rate (FDR) was used to control for Type I error [7] for comparisons involving self-reported lifetime diagnosis. The FDR approach controls for the false positive (or discovery) rate among the tests declared positive or statistically significant, providing a more relevant and accurate criterion for statistical significance. Traditional methods, such as the Bonferroni multiple comparison procedure, become too conservative when the number of tests is large. These methods adjust for the error rate for each comparison by considering all the tests performed rather than only those declared significant. In contrast, the FDR approach considers this information more accurately reflecting the Type I error caused by multiple testing. For the current study, the number of tests used for applying FDR was based on the number of health conditions and the interaction for the overall sample and men and women. Covariates were not counted in determining the number of tests performed since they were not hypothesized as independent variables. FDR significance values (q) are reported as p values in the health condition results.

© 2024 Gasperi M et al. *JAMA Network Open.*

eAppendix.

MVP Program Office

- Sumitra Muralidhar, Ph.D., Program Director
  US Department of Veterans Affairs, 810 Vermont Avenue NW, Washington, DC 20420
- Jennifer Moser, Ph.D., Associate Director, Scientific Programs
  US Department of Veterans Affairs, 810 Vermont Avenue NW, Washington, DC 20420
- Jennifer E. Deen, B.S., Associate Director, Cohort & Public Relations
  US Department of Veterans Affairs, 810 Vermont Avenue NW, Washington, DC 20420

MVP Executive Committee

- Co-Chair: Philip S. Tsao, Ph.D.
  VA Palo Alto Health Care System, 3801 Miranda Avenue, Palo Alto, CA 94304
- Co-Chair: Sumitra Muralidhar, Ph.D.
  US Department of Veterans Affairs, 810 Vermont Avenue NW, Washington, DC 20420
- J. Michael Gaziano, M.D., M.P.H.
  VA Boston Healthcare System, 150 S. Huntington Avenue, Boston, MA 02130
- Elizabeth Hauser, Ph.D.
  Durham VA Medical Center, 508 Fulton Street, Durham, NC 27705
- Amy Kilbourne, Ph.D., M.P.H.
  VA HSR&D, 2215 Fuller Road, Ann Arbor, MI 48105
- Shiuh-Wen Luoh, M.D., Ph.D.
  VA Portland Health Care System, 3710 SW US Veterans Hospital Rd, Portland, OR 97239
- Michael Matheny, M.D., M.S., M.P.H.
  VA Tennessee Valley Healthcare System, 1310 24th Ave. South, Nashville, TN 37212
- Dave Oslin, M.D.
  Philadelphia VA Medical Center, 3900 Woodland Avenue, Philadelphia, PA 19104

MVP Co-Principal Investigators

- J. Michael Gaziano, M.D., M.P.H.
  VA Boston Healthcare System, 150 S. Huntington Avenue, Boston, MA 02130
- Philip S. Tsao, Ph.D.
  VA Palo Alto Health Care System, 3801 Miranda Avenue, Palo Alto, CA 94304

© 2024 Gasperi M et al. *JAMA Network Open.*

MVP Core Operations

- Lori Churby, B.S., Director, MVP Regulatory Affairs
  VA Palo Alto Health Care System, 3801 Miranda Avenue, Palo Alto, CA 94304
- Stacey B. Whitbourne, Ph.D., Director, MVP Cohort Management
  VA Boston Healthcare System, 150 S. Huntington Avenue, Boston, MA 02130
- Jessica V. Brewer, M.P.H., Director, MVP Recruitment & Enrollment
  VA Boston Healthcare System, 150 S. Huntington Avenue, Boston, MA 02130
- Shahpoor (Alex) Shayan, M.S., Director, MVP Recruitment and Enrollment Informatics
  VA Boston Healthcare System, 150 S. Huntington Avenue, Boston, MA 02130
- Luis E. Selva, Ph.D., Executive Director, MVP Biorepositories
  VA Boston Healthcare System, 150 S. Huntington Avenue, Boston, MA 02130
- Saiju Pyarajan Ph.D., Director, Data and Computational Sciences
  VA Boston Healthcare System, 150 S. Huntington Avenue, Boston, MA 02130
- Kelly Cho, M.P.H, Ph.D., Director, MVP Phenomics Data Core
  VA Boston Healthcare System, 150 S. Huntington Avenue, Boston, MA 02130
- Scott L. DuVall, Ph.D., Director, VA Informatics and Computing Infrastructure (VINCI)
  VA Salt Lake City Health Care System, 500 Foothill Drive, Salt Lake City, UT 84148
- Mary T. Brophy M.D., M.P.H., Director, VA Central Biorepository
  VA Boston Healthcare System, 150 S. Huntington Avenue, Boston, MA 02130
- MVP Coordinating Centers
  - MVP Coordinating Center, Boston - J. Michael Gaziano, M.D., M.P.H.
    VA Boston Healthcare System, 150 S. Huntington Avenue, Boston, MA 02130
  - MVP Coordinating Center, Palo Alto – Philip S. Tsao, Ph.D.
    VA Palo Alto Health Care System, 3801 Miranda Avenue, Palo Alto, CA 94304
  - MVP Information Center, Canandaigua – Brady Stephens, M.S.
    Canandaigua VA Medical Center, 400 Fort Hill Avenue, Canandaigua, NY 14424
  - Cooperative Studies Program Clinical Research Pharmacy Coordinating Center, Albuquerque – Todd Connor, Pharm.D.; Dean P. Argyres, B.S., M.S.
    New Mexico VA Health Care System, 1501 San Pedro Drive SE, Albuquerque, NM 87108

MVP Publications and Presentations Committee

- Co-Chair: Themistocles L. Assimes, M.D., Ph. D
  VA Palo Alto Health Care System, 3801 Miranda Avenue, Palo Alto, CA 94304
- Co-Chair: Adriana Hung, M.D.; M.P.H
  VA Tennessee Valley Healthcare System, 1310 24th Ave. South, Nashville, TN 37212
- Co-Chair: Henry Kranzler, M.D.
  Philadelphia VA Medical Center, 3900 Woodland Avenue, Philadelphia, PA 19104

© 2024 Gasperi M et al. *JAMA Network Open.*

MVP Local Site Investigators

- Samuel Aguayo, M.D., Phoenix VA Health Care System
  650 E. Indian School Road, Phoenix, AZ 85012
- Sunil Ahuja, M.D., South Texas Veterans Health Care System
  7400 Merton Minter Boulevard, San Antonio, TX 78229
- Kathrina Alexander, M.D., Veterans Health Care System of the Ozarks
  1100 North College Avenue, Fayetteville, AR 72703
- Xiao M. Androulakis, M.D., Columbia VA Health Care System
  6439 Garners Ferry Road, Columbia, SC 29209
- Prakash Balasubramanian, M.D., William S. Middleton Memorial Veterans Hospital
  2500 Overlook Terrace, Madison, WI 53705
- Zuhair Ballas, M.D., Iowa City VA Health Care System
  601 Highway 6 West, Iowa City, IA 52246-2208
- Jean Beckham, Ph.D., Durham VA Medical Center
  508 Fulton Street, Durham, NC 27705
- Sujata Bhushan, M.D., VA North Texas Health Care System
  4500 S. Lancaster Road, Dallas, TX 75216
- Edward Boyko, M.D., VA Puget Sound Health Care System
  1660 S. Columbian Way, Seattle, WA 98108-1597
- David Cohen, M.D., Portland VA Medical Center
  3710 SW U.S. Veterans Hospital Road, Portland, OR 97239
- Louis Dellitalia, M.D., Birmingham VA Medical Center
  700 S. 19th Street, Birmingham AL 35233
- L. Christine Faulk, M.D., Robert J. Dole VA Medical Center
  5500 East Kellogg Drive, Wichita, KS 67218-1607
- Joseph Fayad, M.D., VA Southern Nevada Healthcare System
  6900 North Pecos Road, North Las Vegas, NV 89086
- Daryl Fujii, Ph.D., VA Pacific Islands Health Care System
  459 Patterson Rd, Honolulu, HI 96819
- Saib Gappy, M.D., John D. Dingell VA Medical Center
  4646 John R Street, Detroit, MI 48201
- Frank Gesek, Ph.D., White River Junction VA Medical Center
  163 Veterans Drive, White River Junction, VT 05009
- Jennifer Greco, M.D., Sioux Falls VA Health Care System
  2501 W 22nd Street, Sioux Falls, SD 57105
- Michael Godschalk, M.D., Richmond VA Medical Center
  1201 Broad Rock Blvd., Richmond, VA 23249
- Todd W. Gress, M.D., Ph.D., Hershel "Woody" Williams VA Medical Center
  1540 Spring Valley Drive, Huntington, WV 25704
- Samir Gupta, M.D., M.S.C.S., VA San Diego Healthcare System
  3350 La Jolla Village Drive, San Diego, CA 92161
- Salvador Gutierrez, M.D., Edward Hines, Jr. VA Medical Center
  5000 South 5th Avenue, Hines, IL 60141

© 2024 Gasperi M et al. *JAMA Network Open.*

- John Harley, M.D., Ph.D., Cincinnati VA Medical Center
  3200 Vine Street, Cincinnati, OH 45220
- Kimberly Hammer, Ph.D., Fargo VA Health Care System
  2101 N. Elm, Fargo, ND 58102
- Mark Hamner, M.D., Ralph H. Johnson VA Medical Center
  109 Bee Street, Mental Health Research, Charleston, SC 29401
- Adriana Hung, M.D., M.P.H., VA Tennessee Valley Healthcare System
  1310 24th Avenue, South Nashville, TN 37212
- Robin Hurley, M.D., W.G. (Bill) Hefner VA Medical Center
  1601 Brenner Ave, Salisbury, NC 28144
- Pran Iruvanti, D.O., Ph.D., Hampton VA Medical Center
  100 Emancipation Drive, Hampton, VA 23667
- Frank Jacono, M.D., VA Northeast Ohio Healthcare System
  10701 East Boulevard, Cleveland, OH 44106
- Darshana Jhala, M.D., Philadelphia VA Medical Center
  3900 Woodland Avenue, Philadelphia, PA 19104
- Scott Kinlay, M.B.B.S., Ph.D., VA Boston Healthcare System
  150 S. Huntington Avenue, Boston, MA 02130
- Jon Klein, M.D., Ph.D., Louisville VA Medical Center
  800 Zorn Avenue, Louisville, KY 40206
- Michael Landry, Ph.D., Southeast Louisiana Veterans Health Care System
  2400 Canal Street, New Orleans, LA 70119
- Peter Liang, M.D., M.P.H., VA New York Harbor Healthcare System
  423 East 23rd Street, New York, NY 10010
- Suthat Liangpunsakul, M.D., M.P.H., Richard Roudebush VA Medical Center
  1481 West 10th Street, Indianapolis, IN 46202
- Jack Lichy, M.D., Ph.D., Washington DC VA Medical Center
  50 Irving St, Washington, D. C. 20422
- C. Scott Mahan, M.D., Charles George VA Medical Center
  1100 Tunnel Road, Asheville, NC 28805
- Ronnie Marrache, M.D., VA Maine Healthcare System
  1 VA Center, Augusta, ME 04330
- Stephen Mastorides, M.D., James A. Haley Veterans' Hospital
  13000 Bruce B. Downs Blvd, Tampa, FL 33612
- Elisabeth Mates M.D., Ph.D., VA Sierra Nevada Health Care System
  975 Kirman Avenue, Reno, NV 89502
- Kristin Mattocks, Ph.D., M.P.H., Central Western Massachusetts Healthcare System
  421 North Main Street, Leeds, MA 01053
- Paul Meyer, M.D., Ph.D., Southern Arizona VA Health Care System
  3601 S 6th Avenue, Tucson, AZ 85723
- Jonathan Moorman, M.D., Ph.D., James H. Quillen VA Medical Center
  Corner of Lamont & Veterans Way, Mountain Home, TN 37684
- Timothy Morgan, M.D., VA Long Beach Healthcare System
  5901 East 7th Street Long Beach, CA 90822
- Maureen Murdoch, M.D., M.P.H., Minneapolis VA Health Care System
  One Veterans Drive, Minneapolis, MN 55417

© 2024 Gasperi M et al. *JAMA Network Open.*

- James Norton, Ph.D., VA Health Care Upstate New York
  113 Holland Avenue, Albany, NY 12208
- Olaoluwa Okusaga, M.D., Michael E. DeBakey VA Medical Center
  2002 Holcombe Blvd, Houston, TX 77030
- Kris Ann Oursler, M.D., Salem VA Medical Center
  1970 Roanoke Blvd, Salem, VA 24153
- Ana Palacio, M.D., M.P.H., Miami VA Health Care System
  1201 NW 16th Street, 11 GRC, Miami FL 33125
- Samuel Poon, M.D., Manchester VA Medical Center
  718 Smyth Road, Manchester, NH 03104
- Emily Potter, Pharm.D., VA Eastern Kansas Health Care System
  4101 S 4th Street Trafficway, Leavenworth, KS 66048
- Michael Rauchman, M.D., St. Louis VA Health Care System
  915 North Grand Blvd, St. Louis, MO 63106
- Richard Servatius, Ph.D., Syracuse VA Medical Center
  800 Irving Avenue, Syracuse, NY 13210
- Satish Sharma, M.D., Providence VA Medical Center
  830 Chalkstone Avenue, Providence, RI 02908
- River Smith, Ph.D., Eastern Oklahoma VA Health Care System
  1011 Honor Heights Drive, Muskogee, OK 74401
- Peruvemba Sriram, M.D., N. FL/S. GA Veterans Health System
  1601 SW Archer Road, Gainesville, FL 32608
- Patrick Strollo, Jr., M.D., VA Pittsburgh Health Care System
  University Drive, Pittsburgh, PA 15240
- Neeraj Tandon, M.D., Overton Brooks VA Medical Center
  510 East Stoner Ave, Shreveport, LA 71101
- Philip Tsao, Ph.D., VA Palo Alto Health Care System
  3801 Miranda Avenue, Palo Alto, CA 94304-1290
- Gerardo Villareal, M.D., New Mexico VA Health Care System
  1501 San Pedro Drive, S.E. Albuquerque, NM 87108
- Agnes Wallbom, M.D., M.S., VA Greater Los Angeles Health Care System
  11301 Wilshire Blvd, Los Angeles, CA 90073
- Jessica Walsh, M.D., VA Salt Lake City Health Care System
  500 Foothill Drive, Salt Lake City, UT 84148
- John Wells, Ph.D., Edith Nourse Rogers Memorial Veterans Hospital
  200 Springs Road, Bedford, MA 01730
- Jeffrey Whittle, M.D., M.P.H., Clement J. Zablocki VA Medical Center
  5000 West National Avenue, Milwaukee, WI 53295
- Mary Whooley, M.D., San Francisco VA Health Care System
  4150 Clement Street, San Francisco, CA 94121
- Allison E. Williams, N.D., Ph.D., R.N, Bay Pines VA Healthcare System
  10,000 Bay Pines Blvd Bay Pines, FL 33744
- Peter Wilson, M.D., Atlanta VA Medical Center
  1670 Clairmont Road, Decatur, GA 30033
- Junzhe Xu, M.D., VA Western New York Healthcare System
  3495 Bailey Avenue, Buffalo, NY 14215-1199

© 2024 Gasperi M et al. *JAMA Network Open.*

- Shing Shing Yeh, Ph.D., M.D., Northport VA Medical Center
  79 Middleville Road, Northport, NY 11768

© 2024 Gasperi M et al. *JAMA Network Open.*

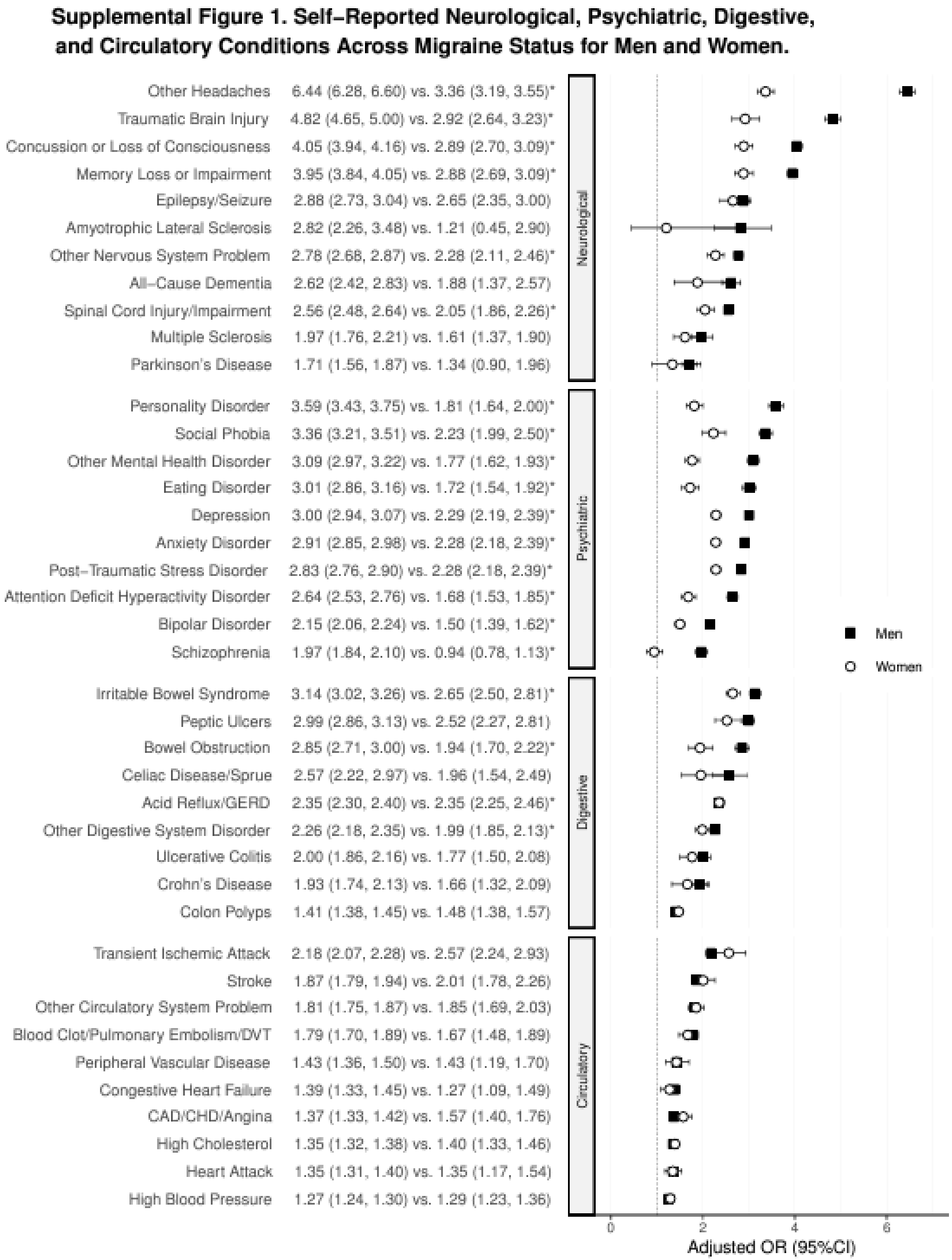

eFigure 1. Self-Reported Neurological, Psychiatric, Digestive, and Circulatory Conditions Across Migraine Status for Men and Women

© 2024 Gasperi M et al. *JAMA Network Open.*

eFigure 2. Self-Reported Musculoskeletal, Hearing and Vision, Infectious, Kidney, Cancer, and Other Conditions Across Migraine Status for Men and Women

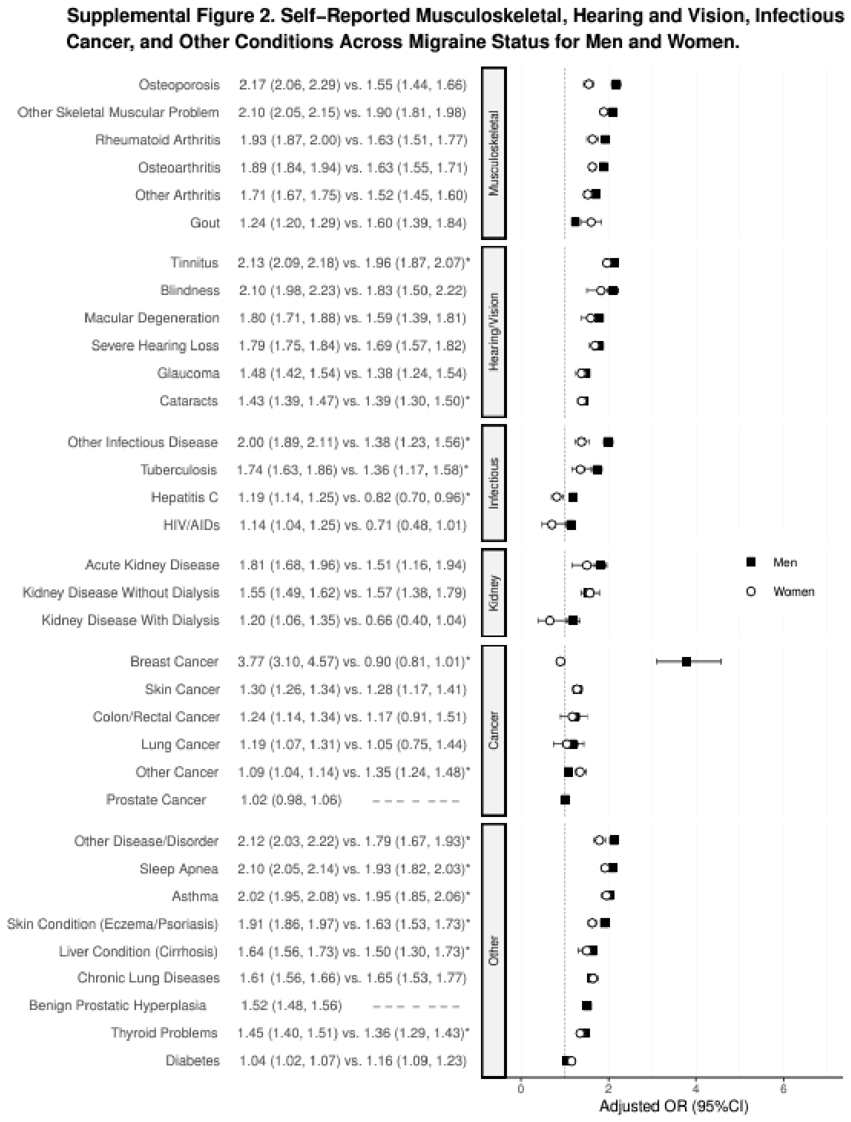

© 2024 Gasperi M et al. *JAMA Network Open.*

eReferences

1. Latinovic R, Gulliford M, Ridsdale L. Headache and migraine in primary care: consultation, prescription, and referral rates in a large population. *J Neurol Neurosurg Psychiatry*. 2006;77(3):385-387.

2. Gaziano JM, Concato J, Brophy M, et al. Million Veteran Program: a mega-biobank to study genetic influences on health and disease. *J Clin Epidemiol*. 2016;70:214-223.

3. Selim AJ, Rogers W, Fleishman JA, et al. Updated US population standard for the Veterans RAND 12-item Health Survey (VR-12). *Qual Life Res*. 2009;18(1):43-52.

4. Palos GR, Mendoza TR, Mobley GM, Cantor SB, Cleeland CS. Asking the community about cutpoints used to describe mild, moderate, and severe pain. *The Journal of Pain*. 2006;7(1):49-56.

5. Proctor S. Development of a structured neurotoxicant assessment checklist (SNAC) for clinical use in veteran populations. *Department of Veterans Affairs, VA Boston Healthcare System*. 2006;

6. Bush K, Kivlahan DR, McDonell MB, Fihn SD, Bradley KA. The AUDIT alcohol consumption questions (AUDIT-C): an effective brief screening test for problem drinking. Ambulatory Care Quality Improvement Project (ACQUIP). Alcohol Use Disorders Identification Test. *Arch Intern Med*. Sep 14 1998;158(16):1789-95. doi:10.1001/archinte.158.16.1789

7. Benjamini Y, Yekutieli D. The control of the false discovery rate in multiple testing under dependency. *Annals of statistics*. 2001:1165-1188.

© 2024 Gasperi M et al. *JAMA Network Open.*
