# Supplementary material for: Migraine Prevalence, Environmental Risk, and Comorbidities in Men and Women Veterans
Source: JAMA Netw Open. 2024 Mar 14;7(3):e242299. doi: 10.1001/jamanetworkopen.2024.2299 (PMC10940970; doi:10.1001/jamanetworkopen.2024.2299)
Supplement: Supplement 3. — Data Sharing Statement [file jamanetwopen-e242299-s003.pdf]

Data Sharing Statement

Gasperi. Migraine Prevalence, Environmental Risk, and Comorbidities in Men and Women Veterans. *JAMA Netw Open*. Published March 14, 2024.
doi:10.1001/jamanetworkopen.2024.2299

Data
Data available: No

Additional Information
Explanation for why data not available: Data will not be made available in order to comply with current VA privacy regulations.
